# Inpatient Outcomes and Complications After Left Atrial Appendage Occlusion in Rural Versus Urban Hospitals in the United States

**DOI:** 10.1002/clc.70397

**Published:** 2026-06-27

**Authors:** Amanda Nguyen, Muhammad Zia Khan, Joseph Neely, Waleed Alruwaili, Muhammad Abdullah Naveed, Justin Lim Devera, William E. Leon, Siddharth Agarwal, Zain Ul Abideen Asad, Samrina Khan, Karthik Gonuguntla, Daniel Ice, Muhammad Bilal Munir

**Affiliations:** ^1^ Department of Medicine University of California Davis Medical Center Sacramento California USA; ^2^ Division of Cardiology Deborah Heart and Lung Center Browns Mills New Jersey USA; ^3^ Department of Medicine West Virginia University Morgantown West Virginia USA; ^4^ Department of Cardiology Dow Medical College Karachi Pakistan; ^5^ Section of Electrophysiology, Division of Cardiology University of California Davis Sacramento California USA; ^6^ Department of Cardiology Mayo Clinic Rochester Minnesota USA; ^7^ Division of Cardiology University of Oklahoma Oklahoma City Oklahoma USA; ^8^ Department of Medicine Khyber Medical University Peshawar Pakistan; ^9^ Division of Cardiology Hartford Hospital Heart and Vascular Institute Norwich Connecticut USA

**Keywords:** disparities, left atrial appendage occlusion, outcomes, rural, urban

## Abstract

**Background:**

Disparities in management of atrial fibrillation in rural and urban hospitals across the U.S. have been reported. However, studies investigating disparities regarding outcomes and complications after left atrial appendage occlusion (LAAO) based on hospital location are lacking.

**Objective:**

To evaluate differences in outcomes and complications related to LAAO among rural and urban hospitals.

**Methods:**

The National Inpatient Sample was used to identify patients who underwent LAAO implantations in the U.S. from 2016−2020. Study endpoints assessed included inpatient complications, outcomes, and resource utilization after LAAO procedures among rural and urban hospitals.

**Results:**

From 2016−2020, there were a total of 87 315 and 1985 LAAO device placements in urban and rural hospitals, respectively. Baseline characteristics were similar among both groups, with a few exceptions. After multivariable adjustment for confounders, odds of inpatient complications and mortality were similar among both groups. However, rural LAAO recipients experienced greater rates of discharge to an acute care facility (3.3% vs 2.4%, *p* = 0.01), as well as lower costs (aOR: 0.58, 95% CI: 0.49–0.69) and length of stay (aOR: 0.89, 95% CI: 0.81–0.98).

**Conclusions:**

A majority of LAAO implantations occurred in urban hospitals in the U.S. Baseline characteristics and adjusted odds of inpatient complications and mortality were similar in rural and urban recipients. However, important differences were described regarding resource utilization and disposition. Further investigation into the specific factors driving these inequities is encouraged to promote improved access to and outcomes of cardiovascular care for rural residents.

## Graphical abstract

A majority of Left atrial appendage occlusion (LAAO) implantations occurred in urban hospitals. Urban patients had higher rates of overall, major, and pulmonary complications, as well as more discharges to home than rural patients in unadjusted analysis.

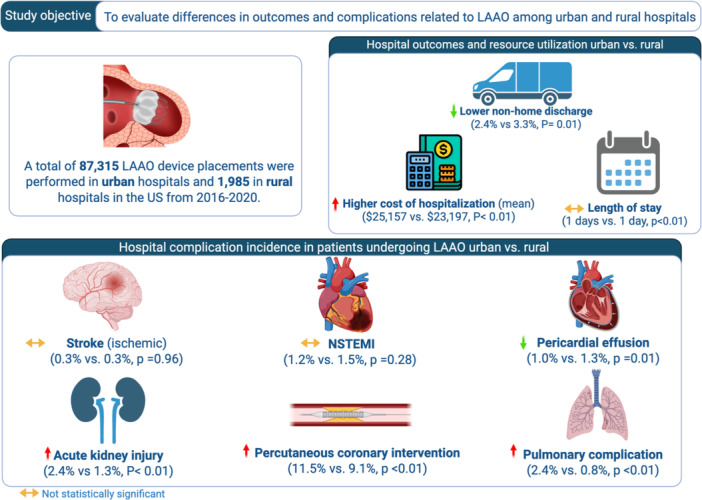

## 1 Introduction

Atrial fibrillation (AF) is associated with increased risk for ischemic stroke, with the majority of cardioembolic strokes originating in the left atrial appendage [1, 2, 3]. In select patients with AF, left atrial appendage occlusion (LAAO) has been shown to be a safe and effective alternative to long‐term oral anticoagulation therapy to mitigate risk of stroke [4, 5, 6, 7, 8]. Although management of AF has evolved significantly over the past few decades due to new pharmaceutical and procedural advancements, certain patient populations fail to benefit from them. In particular, rural patients have been shown to experience lower evaluation rates by cardiovascular specialists, as well as lower utilization rates of guideline directed medical therapies and advanced procedures [9, 10, 11]. Of the rural patients who are able to access cutting edge cardiovascular care, studies have shown that they experience higher complication and mortality rates compared to their urban counterparts [10, 12, 13, 14, 15, 16]. Thus, the purpose of the current study is to assess outcomes and complications of LAAO device implantation in AF patients among rural and urban hospitals.

## 2 Materials and Methods

### 2.1 Data Source

Data from the National Inpatient Sample (NIS) was used for the purpose of our current study. The NIS is a large hospital‐based administrative database that samples inpatient data from 20% of participating hospitals across the nation and is able to estimate > 97% of all U.S. hospitalizations by applying discharge weights. The NIS can be used for computing national estimates of healthcare utilization, costs, trends and outcomes. The database was made possible by a Federal‐State‐Industry partnership sponsored by the Agency for Healthcare Research and Quality (AHRQ) [17]. The data from the NIS is de‐identified, therefore, the need for informed consent and Institutional Review Board approval is waived [17]. The NIS adheres to the 2013 Declaration of Helsinki for the conduct of human research.

### 2.2 Study Population

All percutaneous LAAO device implantations were identified from the NIS database from years 2016–2020 using International Classification of Diseases, 10th Revision, Clinical Modification (ICD‐10‐ CM) code of 02L73DK. The year 2016 was chosen as the start year for the study, as the FDA approved the Watchman device as the first LAAO device in the U.S on March 13, 2015 [18]. Patients younger than 18 years and those with missing demographic data were excluded. The study sample was stratified on the basis of rural versus urban hospital location. As defined by AHRQ, rural and urban hospital location was determined by the Core Based Statistical Area (CBSA), which characterizes micropolitan CBSA and non‐core areas as rural, and metropolitan CBSA as urban [17].

### 2.3 Outcomes

Baseline characteristics, procedural complications, and inpatient outcomes including mortality (reported as a distinct categorical variable in the dataset), length of stay, and hospitalization costs were compared in LAAO device implantations. We also analyzed major complications [defined as composite of pericardial effusion requiring intervention, cardiac arrest, myocardial infarction (NSTEMI & STEMI), ischemic or hemorrhagic stroke, transient ischemia attack, major bleeding, arterial thromboembolism, and peripheral vascular complications (arteriovenous fistula, pseudoaneurysm, access site hematoma, retroperitoneal bleeding, and venous thromboembolism)], inpatient mortality, prolonged hospital stay (defined as length of stay greater than the median length of stay, > 1 day), and increased hospitalization cost (defined as hospitalization cost > median cost $24,752). NIS provides data on total hospital charges. To estimate hospitalization costs, the cost‐to‐charge ratios based upon CMS reimbursement provided by the Healthcare Cost and Utilization Project were applied to the total hospital charges.

### 2.4 Statistical Analysis

Descriptive statistics are presented as frequencies with percentages for categorical variables and as median with inter‐quartile range (IQR) for continuous variables. Baseline characteristics were compared using a Pearson X2 test and Fisher exact test for categorical variables and the Kruskal–Wallis *H* test for continuous variables. For crude comparison of procedural complications and in‐hospital outcomes among the study groups, the Pearson X2 test was used.

To assess the independent association of rural versus urban location with outcomes of mortality, major complications, length of stay and hospitalization costs, a single‐step multivariable logistic regression model was used. Age, sex, race/ethnicity, income, insurance status and selected Elixhauser comorbidities (deficiency anemia, cerebrovascular disorders, congestive heart failure, chronic pulmonary disease, coronary artery disease, diabetes, chronic kidney disease, hypertension, liver disease) were used for adjusted analysis. All these covariates were identified based on prior literature, bivariate analysis and authors best clinical judgement. A *p*‐value of <0.05 was considered statistically significant. All statistical analyses were performed using SPSS version 26 (IBM Corp) and R version 3.6. Because of the complex survey design of the NIS, sample weights, strata, and clusters were applied to raw data to generate national estimates [17].

## 3 Results

A total of 89 300 LAAO device implantations in the U.S. from 2016 to 2020 were identified in our study after applying the relevant exclusion criteria. Of these procedures, 87 315 (97.8%) implantations occurred in urban hospitals, while 1985 (2.2%) occurred in rural hospitals. Baseline characteristics of the study population are shown in Table 1. Urban LAAO recipients tended to be older than their rural counterparts [median age 77 (71–82) vs 75 (71–80), respectively]. The majority of patients undergoing LAAO in rural hospitals were White (99.2% vs 87.4% *p* < 0.01). Overall, the prevalence of co‐morbid conditions was similar between the study groups, with some notable exceptions. Urban patients experienced higher rates of congestive heart failure (35.5% vs 32.7%, *p* < 0.01), chronic kidney disease (24.4% vs 23.9%, *p* < 0.01), and liver disease (2.7% vs 1.3%, *p* = 0.03). Rural patients tended to have lower median income, with more patients in the lowest income quartile (43.8% vs 21.5%, *p* < 0.01), and less patients in the highest income quartile (1.5% vs 24.6%, *p* < 0.01). Furthermore, rural patients were more likely to be insured under public insurance plans like Medicare (88.5% vs 91.2%, *p* < 0.01) and Medicaid (1.2% vs 1.5%, *p* < 0.01) and less likely to have private insurance plans (4.0% vs 8.3%, *p* < 0.01).

**Table 1:** Baseline characteristics in left atrial appendage occlusion device recipients, stratified by rural versus urban hospital location.

Variable no. (%)	Urban *N* = 87315 (97.8%)	Rural *N* = 1985 (2.2%)	*p* value
Age (median [IQR]) years	77 (71–82)	75 (71–80)	< 0.01
Females	36 360 (41.6)	885 (44.6)	< 0.01
Age < 65	6365 (7.3)	155 (7.8)	< 0.01
65–74	27 865 (31.9)	715 (36.0)	
≥75	53 085 (60.8)	1115 (56.2)	
**Race**			
White	73 860 (87.4)	1910 (99.2)	< 0.01
Black	3725 (4.4)	NR (< 11)^a^	
Hispanic	4020 (4.8)	NR (< 11)	
Asian or Pacific Islander	1175 (1.4)	NR (< 11)	
Native American	295 (0.3)	NR (< 11)	
Other	1425 (1.7)	NR (< 11)	
**Co‐morbidities**			
Deficiency anemia	3095 (3.5)	90 (4.5)	0.02
Cerebrovascular disorders	6720 (7.7)	170 (8.6)	0.15
Congestive Heart Failure	31 025 (35.5)	650 (32.7)	0.01
Chronic pulmonary disease	19 510 (22.3)	475 (23.9)	0.09
Coronary artery disease	42 170 (48.3)	945 (47.6)	0.54
Diabetes	15 985 (18.3)	415 (20.9)	< 0.01
Chronic kidney disease	21 340 (24.4)	475 (23.9)	< 0.01
Hypertension	76 005 (87.0)	1760 (88.7)	0.03
Liver disease	2380 (2.7)	25 (1.3)	< 0.01
Obesity	15 130 (17.3)	465 (23.4)	< 0.01
Peripheral vascular disorders	7715 (8.8)	160 (8.1)	0.23
Hypothyroidism	15 705 (18.0)	320 (16.1)	0.03
Smoking status	3835 (4.4)	70 (3.5)	0.06
**Median income**			
0–25th	18 535 (21.5)	850 (43.8)	< 0.01
26–50th	22 560 (26.2)	780 (40.2)	
51–75th	23 890 (27.7)	280 (14.4)	
76–100th	21 145 (24.6)	30 (1.5)	
**Insurance**			
Medicare	77 215 (88.5)	1810 (91.2)	< 0.01
Medicaid	1090 (1.2)	30 (1.5)	
Private insurance	7250 (8.3)	80 (4.0)	
Self‐pay	350 (0.4)	NR (< 11)	
No charge	30 (0)	NR (< 11)	
Other	1305 (1.5)	55 (2.8)	
**Region**			
Northeast	5370 (6.2)	55 (2.8)	< 0.01
Midwest	7110 (8.1)	445 (22.4)	
South	13 250 (15.2)	105 (5.3)	
West	8115 (9.3)	25 (1.3)	

^a^ Less than 11 data were not reported as per HCUP recommendations.

Other important procedure related complications and inpatient outcomes after LAAO and stratified on the basis of hospital location are shown in Tables 2 and 3, respectively. The prevalence of overall complications, major complications, pulmonary complications, and acute kidney injury was higher in urban patients compared to their rural counterparts [(9.4% vs. 8.1%, *p* = 0.04), (5.9% vs 4.5%, *p* = 0.01), (2.4% vs 0.8%, *p* < 0.01), and (2.4% vs 1.3%, *p* < 0.01), respectively]. Inpatient mortality was low among both rural and urban patients. Rural patients were found to have more non‐home discharges to acute care facilities (3.3% vs 2.4%, *p* = 0.01) and lower cost of hospitalization ($23 197.67 vs $25 157.48, *p* < 0.01).

**Table 2:** Left atrial appendage closure device complications, stratified by rural vs urban hospital location.

Variables no. (%)	Urban *N* = 87315 (97.8%)	Rural *N* = 1985 (2.2%)	*p* value
**Overall complications (%)**	8195 (9.4)	160 (8.1)	0.04
**Major complications (%)** a	5145 (5.9)	90 (4.5)	0.01
**Any cardiovascular event/complication**	2365 (2.7)	65 (3.3)	0.12
Percutaneous coronary intervention	10 070 (11.5)	180 (9.1)	< 0.01
Cardiac Arrest/CPR procedure code	115 (0.1)	NR (< 11)^b^	< 0.01
Pacemaker implantation	320 (0.4)	NR (< 11)	0.4
STEMI	40 (0.1)	NR (< 11)	0.34
NSTEMI or type II MI	1085 (1.2)	30 (1.5)	0.28
Pericardial effusion requiring intervention	865 (1.0)	25 (1.3)	< 0.01
Tamponade	595 (0.7)	NR (< 11)	0.02
Pericarditis	145 (0.2)	NR (< 11)	0.07
Cardiogenic Shock	185 (0.2)	NR (< 11)	0.7
**Any systemic complication**	130 (0.1)	NR (< 11)	0.24
Anaphylaxis	25 (0.0)	NR (< 11)	0.4
Arterial embolism	75 (0.1)	NR (< 11)	0.19
Septic Shock	35 (0.0)	NR (< 11)	< 0.01
**Any peripheral vascular complication**	1655 (1.9)	30 (1.5)	0.21
AV fistula	145 (0.2)	NR (< 11)	0.07
Pseudoaneurysm	290 (0.3)	NR (< 11)	0.01
Hematoma	440 (0.5)	NR (< 11)	1
Retroperitoneal Bleeding	60 (0.1)	NR (< 11)	0.23
Venous thromboembolism	225 (0.3)	NR (< 11)	0.03
**Any neurological complication**	655 (0.8)	NR (< 11)	0.21
Hemorrhagic stroke	250 (0.3)	5 (0.3)	0.76
Ischemic stroke	225 (0.3)	5 (0.3)	0.96
TIA	200 (0.2)	NR (< 11)	0.03
Any GI or hematological complication/bleeding	50 (0.1)	NR (< 11)	0.47
GI bleeding	3350 (3.8)	70 (3.5)	< 0.02
Bleeding during the procedure	65 (0.1)	NR (< 11)	< 0.01
Need for blood transfusion	1360 (1.6)	60 (3.0)	< 0.01
**Any Pulmonary complications**	2130 (2.4)	15 (0.8)	< 0.01
Respiratory failure	1020 (1.2)	NR (< 11)	< 0.01
Pneumothorax	25 (0.0)	NR (< 11)	< 0.01
Pleural Effusion	325 (0.4)	NR (< 11)	< 0.01
Pneumonia bacterial	240 (0.3)	NR (< 11)	0.02
Ventilation	95 (0.1)	NR (< 11)	0.14
Acute Kidney injury	2135 (2.4)	25 (1.3)	< 0.01

^a^ Composite of cardiac arrest, ischemic stroke, hemorrhagic stroke, TIA, arterial embolism, myocardial infarction (NSTEMI & STEMI) major bleeding, pericardial effusion requiring intervention and peripheral vascular complications.

^b^ Less than 11 data were not reported as per HCUP recommendations.

**Table 3:** Hospital outcomes and resource utilization in left atrial appendage occlusion device recipients, stratified by rural vs urban hospital location.

Variables no. (%)	Urban *N* = 87315 (97.8%)	Rural *N* = 1985 (2.2%)	*p* value
Died at discharge	135 (0.2)	NR (< 11)	0.26
Home/Routine/self‐care	85 060 (97.6)	1890 (96.7)	0.01
Non‐home discharges	2120 (2.4)	65 (3.3)	
Length of stay, days	1(1–1)	1 (1–1)	< 0.01
Cost of hospitalization, $	25 157.48 (19 439.12–31 928.14)	23 197.67 (18 320.09–30 329.9)	< 0.01

**Figure 1:**
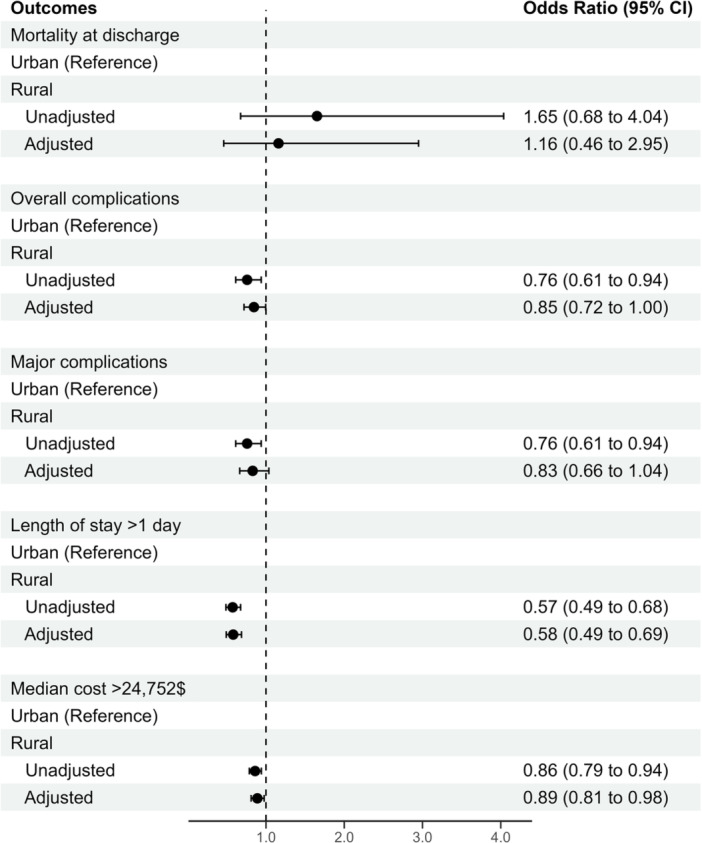
Adjusted association of rural and urban hospital location with outcomes of mortality, major complications, prolonged length of stay and increased hospitalization costs.

Multivariable models adjusting for potential confounders were constructed to assess the independent association of hospital location with outcomes after LAAO implantation (Figure 1). After adjustment, odds of inpatient mortality, overall complications, and major complications were similar among rural and urban patients [(aOR: 1.16, 95% CI: 0.46–2.95), (aOR: 0.85, 95% CI: 0.72–1.00), and (aOR: 0.83, 95% CI: 0.66–1.04), respectively]. Rural patients undergoing LAAO had decreased length of stay (aOR: 0.58, 95% CI: 0.49–0.69) and decreased cost of hospitalization (aOR: 0.89, 95% CI: 0.81–0.98).

## 4 Discussion

In this large, contemporary, real‐world cohort of LAAO implantations in the US, we report several main findings: (1) a majority of LAAO implantations occurred in urban hospitals (2) urban patients had higher rates of overall, major, and pulmonary complications than rural patients, but there was no significant difference after adjusting for potential confounding variables (3) rural patients had more non‐home discharges to an acute care facility after LAAO implantation compared to their urban counterparts (4) rural hospital location was independently associated with decreased length and cost of stay after LAAO implantation.

Health disparities among rural patients have been well documented within medicine, including cardiology. Rural patients have been shown to have lower rates of advanced cardiac procedures, worse procedural outcomes, increased complication rates and increased inpatient mortality [10, 12, 13, 14, 15, 16, 19, 20, 21]. Although rural individuals make up approximately 20% of the U.S. population, rural patients made up only 2.2% of the patient population in our study cohort [22]. The low rates of LAAO implantation among rural patients are likely related to a multitude of factors, such as transportation barriers and limited access to or referral to higher level of care. This is a common theme that has been demonstrated among advanced cardiac procedures such as transcatheter aortic valve replacement, implantable cardioverter‐defibrillators and pacemaker implantations, and catheter ablations [11, 23, 24, 25, 26]. Challenges in recruiting, training, and retaining specialists in rural settings also likely contribute to this issue [27, 28]. When exploring patient perspectives, various studies have also reported higher refusal rates for advanced cardiac procedures among rural patients [14, 25, 29]. This may be related to differences in health education, literacy and beliefs, and could serve as an area for intervention.

Moreover, rural patients often experience greater socioeconomic disparities than their urban counterparts, further driving health care inequities [11, 14, 20, 23, 24, 25, 28]. In our cohort, rural patients tended to be in lower median income quartiles than their urban counterparts. In addition, rural patients were more likely to be insured under public insurances policies, while urban patients were more often insured under private insurance policies. This could potentially influence the ability for rural patients to dedicate time, resources and money to schedule and recover from a specialized cardiac procedure such as LAAO.

In contrast to prior studies that demonstrated higher complication and mortality rates in rural hospital patients undergoing various cardiac interventions, we found that urban patients had higher rates of overall and major complications than rural patients in unadjusted analysis [10, 13, 16, 19, 20]. One explanation for this could be related to higher baseline acuity and/or severity of patients that selectively present to urban hospitals. For example, perhaps the highest‐risk rural patients were referred to undergo LAAO at high‐volume, tertiary‐care urban hospitals due to underlying comorbidities. Consequently, after adjusting for potential confounding variables including common co‐morbidities, there was no difference in odds of overall and major complications, as well as inpatient mortality among rural and urban patients. Given that prior studies have shown lower procedural rates, higher complications and mortality in Black, Hispanic and lower socioeconomic patients after LAAO, our multivariable regression also adjusted for race/ethnicity, income, and insurance status [30, 31, 32, 33, 34].

Rural patients undergoing LAAO in our study did have lower cost and length of stay, before and after adjustment. This may be related to reimbursement differences that affect rural hospitals, with rural hospitals often being reimbursed less from public insurance plans than urban hospitals for the same service. The better outcomes for resource utilization among rural patients could be related to the lower rates of complications observed in our study. However, we did find that rural patients undergoing LAAO had higher discharge rates to acute care facilities, suggesting differences in post‐procedure recovery.

In summary, our study contributes to the growing evidence base on health care disparities experienced by rural patients. Although only a minority of rural patients underwent LAAO compared to their urban counterparts, procedural outcomes and inpatient mortality was similar after adjusting for confounders. Important differences in resource utilization and disposition were described.

## 5 Limitations

The results of our current study should be interpreted in the context of the following limitations. First, the NIS relies on administrative coding for disease and procedure identification which may be subject to errors. However, it should be noted that the NIS uses a rigorous data quality control program to minimize miscoding and ensure integrity of data. Second, as the NIS includes data on inpatient stays only, long‐term outcomes and mortality cannot be ascertained. Third, there is no data available on procedural characteristics, such as use of contrast, length of procedure, operator experience, or volume of device placement per hospital. Additionally, as our study spans several years from 2016 to 2020, there were likely improvements in operator experience, particularly in rural regions, that may be affecting study outcomes. Fourth, there is no data available on procedural success, such as reduction in incidence of stroke. Fifth, although we adjusted for various demographic and clinical variables, the NIS provides limited granularity on social determinants of health, and other socioeconomic or structural inequities likely persist that remain unaccounted for in our analysis. Sixth, due to sample size constraints, absolute number of events in rural patients were limited if the event size was <11. Thus, granular detail regarding procedural outcomes and events could not be obtained in these circumstances.

## 6 Conclusions

In this large, contemporary, real‐world cohort of LAAO implantations in the U.S., rural patients had lower overall procedural rates compared to urban patients. After adjusting for important confounding variables, rural and urban patients undergoing LAAO had similar inpatient outcomes and mortality rates. Interestingly, rural patients generally had lower complication rates and resource utilization, but greater discharges to acute care facilities. Further investigation into the root cause of these disparities is essential to strive for more equitable access to and outcomes of advanced cardiovascular care.

## Author Contributions

Amanda Nguyen was involved in conceptualization, methodology, formal analysis, and writing the original draft. Muhammad Zia Khan was involved in conceptualization, data curation, methodology, formal analysis, and reviewing and editing the draft. Waleed Alruwaili was involved in visualization and reviewing and editing the draft. Joseph Neely, Justin Lim Devera, William E. Leon, Siddharth Agarwal, Zain Ul Abideen Asad, Samrina Khan, Karthik Gonuguntla, Daniel Ice were involved in conceptualization and reviewing and editing the draft. Muhammad Bilal Munir was involved in conceptualization, methodology, supervision, and reviewing and editing the draft.

## Funding

The authors have nothing to report.

## Conflicts of Interest

The authors declare no conflicts of interest.

## Data Availability

The data that support the findings of this study are available in HCUP at https://hcup-us.ahrq.gov/nisoverview.jsp. These data were derived from the following resources available in the public domain‐ HCUP,https://hcup-us.ahrq.gov/db/nation/nis/nisdbdocumentation.jsp.
